# Neurological outcome in patients after successful resuscitation in out-of-hospital settings

**DOI:** 10.17305/bjbms.2020.4623

**Published:** 2020-08

**Authors:** Martin Marinšek, Andreja Sinkovič, David Šuran

**Affiliations:** 1 Department of Medical Intensive Care, University Clinical Centre Maribor, Maribor, Slovenia; 2 Medical Faculty, University of Maribor, Maribor, Slovenia; 3 Department of Cardiology and Angiology, University Clinical Centre Maribor, Maribor, Slovenia

**Keywords:** Out-of-hospital cardiac arrest, OHCA, ischemic brain injury, resuscitation

## Abstract

Neurological outcome is an important determinant of death in admitted survivors after out-of-hospital cardiac arrest (OHCA). Studies demonstrated several significant pre-hospital predictors of ischemic brain injury (time to resuscitation, time of resuscitation, and cause of OHCA). Our aim was to evaluate the relationship between post-resuscitation clinical parameters and neurological outcome in OHCA patients, when all recommended therapeutic strategies, including hypothermia, were on board. We retrospectively included consecutive 110 patients, admitted to the medical ICU after successful resuscitation due to OHCA. Neurological outcome was defined by cerebral performance category (CPC) scale I-V. CPC categories I-II defined good neurological outcome and CPC categories III-V severe ischemic brain injury. Therapeutic measures were aimed to achieve optimal circulation and oxygenation, early percutaneous coronary interventions (PCI) in acute coronary syndromes (ACS), and therapeutic hypothermia to improve survival and neurological outcome of OHCA patients. We observed good neurological outcome in 37.2% and severe ischemic brain injury in 62.7% of patients. Severe ischemic brain injury was associated significantly with known pre-hospital data (older age, cause of OHCA, and longer resuscitations), but also with increased admission lactate, in-hospital complications (involuntary muscular contractions/seizures, heart failure, cardiogenic shock, acute kidney injury, and mortality), and inotropic and vasopressor support. Good neurological outcome was associated with early PCI, dual antiplatelet therapy, and better survival. We conclude that in OHCA patients, post-resuscitation early PCI and dual antiplatelet therapy in ACS were significantly associated with good neurological outcome, but severe ischemic brain injury was associated with several in-hospital complications and the need for vasopressor and inotropic support.

## INTRODUCTION

The incidence of out-of-hospital cardiac arrest (OHCA) in Europe is on average 84 cases/100.000 population/year with achieved return of spontaneous circulation (ROSC) in 9–50% of resuscitation attempts and survival-to-discharge of admitted patients in 6.4–66.9%. The majority of admitted OHCA patients are usually comatose, intubated, and mechanically ventilated [1].

According to previous studies, 24 hours of mild therapeutic hypothermia with target temperatures of 32–34°C, as well as temperatures up to 36°C, improved neurological outcome [2,3].

Both ischemic brain injury and cardiogenic shock are the most significant predictors of short-term mortality [4,5]. However, neurological status alone is the major determinant of the long-term prognosis [6,7]. Neurological impairment includes cognitive impairment, restricted mobility, depression, and even vegetative state or brain death in survivors of OHCA [8,9]. Post-cardiac arrest brain injury is most widely assessed by the cerebral performance category (CPC) scale and corresponds to the quality of life and outcome [9]. CPC categories I and II represent good neurological outcome. Patients in CPC category I are conscious, alert, and able to work and lead a normal life as their psychological or neurologic deficits are minor. Patients in CPC category II are conscious but able to work part-time in a sheltered environment with independent activities of daily life; moderate neurological deficits may be present [8,9]. Severe brain injury includes CPC categories III-V. Patients in CPC category III are conscious, dependent on daily support in an institution or at home with exceptional family effort, with limited cognition and significant neurological deficits. CPC category IV includes patients in a vegetative state and CPC category V includes patients with brain death or death by the traditional criteria, who never gained consciousness in the post-resuscitation period [8,9].

Currently, in unconscious patients, neurological prognostication is not recommended earlier than 72 hours after OHCA [10-12]. In the early post-resuscitation period, soon after cessation of analgesia and sedation, suspicion of severe neurological dysfunction is raised by the absence of corneal and pupillary reflexes, myoclonus within the next few days, epileptic status, registered by electroencephalogram (EEG), diffuse anoxic changes on computed tomography/magnetic resonance (CT/MR), and increased levels of the brain injury markers neuron-specific enolase and protein S-100 [13]. However, according to previous trials, the most significant independent predictors of neurological dysfunction after OHCA are prehospital data such as asystole/pulseless electrical activity (PEA) as the cause of OHCA, long resuscitation time in addition to the absence of corneal and pupillary reflexes, absence of motoric response on admission, adrenalin therapy, metabolic acidosis, and admission arterial PCO_2_ < 4.5 kPa [12,14]. Our aim was to evaluate the relationship between clinical parameters of the post-resuscitation period and neurological outcome in OHCA patients, when all recommended therapeutic strategies, including hypothermia, were on board.

## MATERIALS AND METHODS

We retrospectively included 110 patients, successfully resuscitated in the out-of-hospital settings and admitted alive to the Department of Medical Intensive Care Unit (MICU) at the tertiary University Clinical Centre Maribor (Slovenia) from 2014 to 2016 (72.7% men, mean age 65.6 ± 13.8 years, age ≥65 years in 72.7% of patients).

In the out-of-hospital settings, witnesses of OHCA usually activated emergency medical services (EMS) team by calling the emergency number 112 of the emergency communication center. Often, the witness started basic life support in victims of OHCA (chest compressions), until the arrival of the EMS team. The EMS team confirmed the cardiac arrest, and attached the monitor and performed defibrillation in case of ventricular fibrillation (VF) or pulseless ventricular tachycardia (VT). In nonresponders and in case of asystole or PEA, the EMS continued chest compressions and administered adrenalin. In the case of prolonged resuscitation with respiratory arrest the patients were intubated and mechanically ventilated. In case of ROSC, the patients were transported to the MICU. The resuscitation time of the EMS team was registered in minutes.

On arrival, the majority of OHCA patients were comatose, but with palpable pulse, and the majority were intubated and mechanically ventilated [10,11].

After arrival to the MICU, all OHCA patients were monitored by continuous ECG, pulse oximetry, continuous systemic arterial blood pressure, and intermittent central venous pressure measurements. Blood samples were drawn to measure standard laboratory tests. Echocardiography was performed to measure ejection fraction (EF) and left ventricular end-diastolic diameter, and to evaluate valves, possible pericardial effusion or pleural pathologies [10,11].

The goal of post-resuscitation care was to achieve adequate oxygenation by mechanical ventilation (MV), adequate circulation by infusion of fluids, intravenous (IV) vasopressors and/or inotropes and/or mechanical circulatory support by insertion of an intra-aortic balloon pump (IABP) [11]. Post-resuscitation care protocol was in agreement with the guidelines. It included oxygenation targets (SatO_2_ 90–95%, arterial pO_2_ 8.5–9 kPa, arterial pCO_2_ 5–6 kPa, and normal pH), serum lactate <2 mmol/l, ventilation targets (tidal volumes 6–8 ml/ideal body weight), and circulatory targets (mean arterial pressure >70 mmHg, heart rate 60–100/min, and ScvO_2_ > 70%) [10,11].

We controlled involuntary muscular contractions/seizures by anticonvulsant drugs (benzodiazepines, levetiracetam, and barbiturates), hyperglycemia by short-acting insulin infusion and concomitant blood glucose measurements per 2–3 hours with target blood glucose level of 6–11 mmol/l, avoiding hypoglycemia. We controlled arrhythmias by antiarrhythmic drugs [10,11].

Acute coronary syndromes (ACS) were confirmed by standard ECG recordings, patient history, and troponin I measurements, followed by coronary angiography and percutaneous coronary intervention (PCI), if necessary, combined with dual antiplatelet therapy, including acetylsalicylic acid (ASA) and P2Y12 inhibitors [6,11,15].

We started targeted temperature management to prevent ischemic brain injury [2,16]. Target temperatures, measured by urine catheter, were 32–34°C. They were achieved by a rapid infusion of 20–30 ml/kg of cold saline (4°C) over 20–30 minutes. Induction time in patients was approximately 60 minutes. The amount of infused cold saline was adjusted to worsening respiratory failure and/or pulmonary edema if present [6,10,11]. The core temperature of 32–34°C, if reached, was maintained for the next 24 hours by cooled blankets (CritiCool^®^ device, Israel) [6]. Hypothermia was followed by gradual, 6–8 hour, rewarming till normothermia [6,10,11].

The underlying disease of OHCA was categorized as ACS, chronic ischemic coronary heart disease without coronary occlusion or stenosis, or nonischemic heart disease (valvular, cardiomyopathies, pulmonary embolism, and acute heart failure) as assessed by echocardiography and troponin measurements.

Clinical and neurological assessments were performed each day by the treating intensivist. However, prognostic assessment of neurological status was started more than 72 hours after admission by an experienced neurologist, performing neurological clinical examination and additional diagnostics such as EEG and CT/MR. Neurological examination was repeated, if necessary. The most important measure to report neurological outcome was assessment of CPC categories I-V, where CPC categories I and II determined good neurological outcome and CPC categories III-V ischemic brain injury [8,9].

Survivors with good neurological outcome were candidates for preventive measures of recurrent cardiac arrest such as implantable cardioverter-defibrillator (ICD), cardiac pacing, ectopic focus ablation, and valve replacement [10,11].

In all the included OHCA patients, we registered age (≥65 years), gender, admission lactate levels (≥6 mmol/L), the incidence of VF/VT or asystole/PEA as the cause of OHCA, resuscitation time ≥20 minutes, admission EF <35%, ACS as the underlying disease, admission and in-hospital MV, in-hospital treatments by PCI, mild therapeutic hypothermia of 32–34°C, and the need for dobutamine, noradrenalin, IABP, and antibiotics. Among in-hospital complications, in addition to CPC categories I-V, we registered the incidence of cardiogenic shock, arrhythmias, bleedings, infections, involuntary muscular contractions/seizures, and acute kidney injury (AKI) at any time of in-hospital stay, as well as in-hospital mortality.

Arrhythmias were registered by continuous ECG monitoring and standard ECG recordings and were classified as atrial, ventricular, or conduction disturbances. Shock was quantified clinically by the need for noradrenalin and/or inotropic support to maintain adequate circulation and systolic function [17]. Infection was defined as the presence of microorganisms in otherwise sterile milieu of the body (blood, cerebrospinal liquor, lung tissue, and urinary tract) with or without clinical symptoms (fever, increased C-reactive protein [CRP], leukocytosis, or leukocytopenia), or with antibiotic administration due to strong clinical suspicion of infection [18].

According to the Thrombolysis In Myocardial Infarction (TIMI) criteria, major bleeding was defined as cerebral or symptomatic bleeding in other locations with a hemoglobin level drop of >50 g/l or the need for ≥2 units of blood product transfusions. Minor bleeding was defined as symptomatic with a hemoglobin level drop 30–50 g/L. Minimal bleeding was defined as symptomatic with a hemoglobin level drop <30 g/l [19].

AKI was defined as an increase in serum creatinine of at least 50% within 24–48 hours [20].

In case of complications, patients were treated according to professional protocols by the discretion of the treating physician (e.g., by vasopressors, inotropic agents, MV, IABP, red blood cell transfusions, antibiotics, antiarrhythmic drugs, and pacing) [10,11].

Finally, we compared baseline characteristics, laboratory and clinical data, treatments, and outcome between patients with good neurological outcome (CPC categories I and II) and patients with severe brain injury (CPC categories III-V).

### Ethical statement

The Institutional Medical Ethics Committee (UKC-KME-02/19) approved the study. As this was a retrospective, single-center observational study, the need for informed consent was waived. Personal data of all the patients were protected according to the Law on personal data protection.

### Statistical analysis

Statistical analysis was performed using IBM SPSS Statistics for Windows, Version 24.0. (IBM Corp., Armonk, NY, USA). Data are expressed as means ± standard deviations or percentages. Differences between the groups were tested by the two-sided Student’s t-test for means ± standard deviations and by the Chi-square test for percentages.

Kaplan–Meier survival plot was employed to analyze association of hospital survival with neurological status on discharge. A value of *p* < 0.05 was considered statistically significant.

## RESULTS

Baseline clinical data are presented in Table 1. Age ≥65 years was observed in 41.8% of all OHCA patients. The majority of admitted OHCA patients were in coma (87.3%) and mechanically ventilated (87.3%). VF/VT was the cause of OHCA in 63.6% and asystole/PEA in 35.5% of patients. OHCA was witnessed in 73.6% of patients. Resuscitation ≥20 minutes by EMS was observed in 44.5% of cases. On admission, we observed lactate levels ≥6 mmol/L in 30%, and EF <35%, measured by echocardiography, in 43.6% of cases. We diagnosed ACS on admission in 55.4% of OHCA patients, including ST-elevation myocardial infarction (STEMI) in 43.6% and non-STEMI (NSTEMI) in 11.8%.

**TABLE 1:**
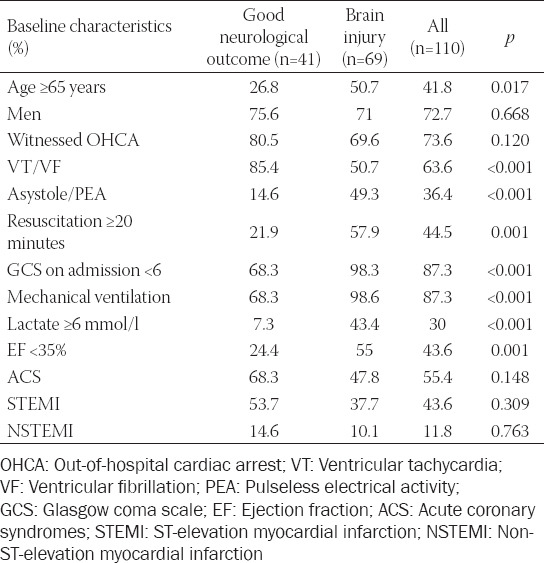
Baseline characteristics

In-hospital treatments are listed in Table 2. We reached target temperatures of 32–34°C by mild therapeutic hypothermia within the first few hours in 71.8% and performed PCI in 40% of cases; OHCA patients received IV noradrenalin infusion in 75.5%, IV inotropic support in 42.7% (dobutamine in 29.1% and levosimendan in 18.2%), IABP in 3.6%, antibiotics in 76.4%, and in-hospital MV in 88.2% of cases.

**TABLE 2:**
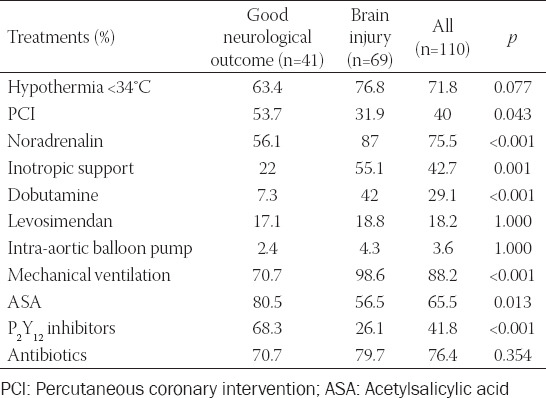
In-hospital treatments

In-hospital complications are presented in Table 3. We observed bleedings in 10%, infection in 70.9%, AKI in 32.7%, involuntary muscular contractions/seizures in 27.2%, in-hospital heart failure of Killip classes II-IV in 82.7%, cardiogenic shock in 75.5%, arrhythmias in 32.7%, and severe ischemic brain injury (CPC categories III-V) in 62.7% of cases, whereas good neurological outcome in 37.3% of OHCA patients and in-hospital mortality in 51.8%.

**TABLE 3:**
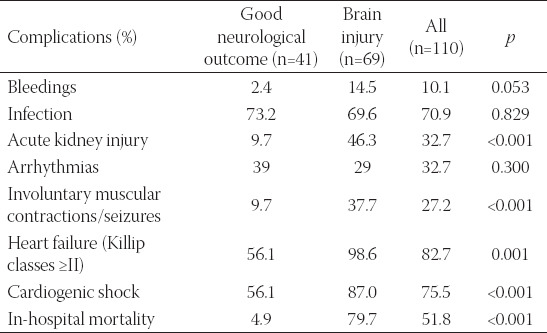
In-hospital complications

Severe brain injury (CPC categories III-V) in comparison to good neurological outcome (CPC categories I-II) in OHCA patients was associated significantly more likely with age ≥65 years, asystole/PEA as the cause of OHCA, resuscitation ≥20 minutes, MV on admission and during in-hospital stay, admission lactate ≥6 mmol/l, EF <35%, and Glasgow Coma Scale (GCS) <6 (Table 1). OHCA patients with severe brain injury (CPC categories III-V) in comparison to patients with good neurological outcome (CPC categories I-II) were significantly more likely treated in the MICU by vasopressors, inotropic support by dobutamine, and more likely by MV. There was no significant difference in the use of therapeutic hypothermia between patients with and without brain injury (Table 2). In elderly (≥65 years of age) and younger OHCA patients, hypothermia of 32–34°C was achieved at a similar rate (73.9% vs. 70%).

Severe brain injury (CPC categories III-V) in comparison to good neurological outcome (CPC categories I-II) in OHCA patients was associated significantly with AKI, involuntary muscular contractions/seizures, heart failure, in particularly shock, and hospital mortality (Table 2).

Good neurological outcome was associated significantly with VT/VF as the immediate cause of OHCA, treatment by PCI, ASA and P_2_Y_12_ inhibitors, and less likely with complications and treatment by inotropic and vasopressor support (Table 1-3).

**FIGURE 1:**
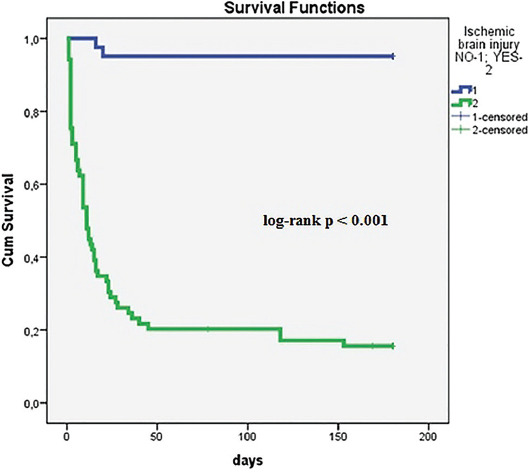
Kaplan–Meier survival curves in patients with ischemic brain injury and good neurological outcome after OHCA. Kaplan–Meier survival plot in green represents survival with ischemic brain injury and in blue survival with good neurological outcome. At censoring, 95.1% of OHCA patients with good neurological outcome were alive at hospital discharge compared with 20.3% of those with ischemic brain injury (log-rank *p* < 0.0015). OHCA: Out-of-hospital cardiac arrest.

Kaplan–Meier survival plot is displayed in Figure 1. At censoring, 95.1% of OHCA patients with good neurological outcome were alive at hospital discharge compared with 20.3% of those with ischemic brain injury (log-rank *p* < 0.0015).

## DISCUSSION

In our retrospective analysis of OHCA patients, who were admitted alive to the MICU, we observed severe ischemic brain injury (CPC categories III-V) in 62.7% and good neurological outcome in 37.3% of cases. Ischemic brain injury was associated significantly with asystole/PEA as the cause of OHCA, resuscitation ≥20 minutes, age ≥65 years, admission EF <35% and lactate ≥6 mmol/L, GCS <6, involuntary muscular contractions/seizures, and with cardiogenic shock. Good neurological outcome was associated significantly with PCI treatment, and the use of ASA and P_2_Y_12_ inhibitors.

According to the EuReCa ONE study, only approximately 5% of patients gained full neurological recovery [1]. In the last decade in our environment, approximately 50% of OHCA patients, who were resuscitated, gained ROSC in the out-of-hospital settings [6,21]. Their overall good neurological outcome was achieved in approximately 13% cases between 2001 and 2004 and in 17% cases between 2011 and 2013 [6,21]. In the present retrospective analysis from 2014 to 2016, 37.3% of OHCA patients with ROSC on admission to the MICU gained full neurological recovery, which represents overall good neurological outcome in 18% of cases.

In OHCA patients admitted alive to the MICU, previous studies demonstrated that adequate post-resuscitation care, including the maintenance of adequate circulation, ventilation, oxygenation, and early treatment of ACS by PCI in addition to therapeutic hypothermia, has a significant potential to decrease the incidence of ischemic brain injury [15,22]. Therefore, these measures are implemented in the contemporary guidelines for post-resuscitation care and were fulfilled in our patients as well [10,11].

Severe ischemic brain injury, usually categorized as CPC III-V, is a major determinant of long-term prognosis in OHCA patients. Among post-resuscitation care measures, therapeutic hypothermia has the potential to prevent it, according to previous studies [2,16]. Therapeutic hypothermia, which is incorporated in post-resuscitation care of OHCA patients, exerts several benefits at the cellular level. It reduces cerebral metabolism and lactate production; it reduces oxygen demand, improves cerebral energy stores and the use of glucose; and it decreases inflammation and production of free radicals [7].

In our OHCA patients, induction of hypothermia was started within the first few hours of in-hospital stay, most preferably within the first 1–2 hours. Hypothermia of 32–34°C was achieved in 71.8% of all OHCA patients, less in those with very early gain of consciousness and early death. Between patients with good and adverse neurological outcome, we did not observe any significant differences in the achieved target temperatures 32–34°C. This finding is in agreement with a large study demonstrating that therapeutic normothermia or temperature control at 36°C may be equally effective as hypothermia at 32–34°C in long-term regarding neurological outcomes in brain injury [3,23]. On the other hand, the interaction of different variables such as age and/or other unidentified processes determine the magnitude by which hypothermia increases the activation of cold stress molecules in biological systems, which alter brain physiology during therapeutic hypothermia. Therapeutic hypothermia is robustly influenced by age and is beneficial more in younger patients in addition to other therapeutic measures in the ICU [23].

Brain tissue is particularly vulnerable not only to ischemia during OHCA but also to secondary injury occurring in post-resuscitation period, due to imbalance in post-resuscitation cerebral oxygen delivery and microcirculatory reperfusion injury, endothelial dysfunction, free radical formation, and intracellular calcium accumulation [7].

Ischemic brain injury is a multifactorial complication in OHCA patients admitted to the MICU alive. Studies demonstrated that a strong predictor of good neurological outcome and survival was VF/VT as the immediate cause of OHCA, in addition to cardiac etiology of VF/VT and resuscitation <20 minutes [14]. In our patients, coronary artery disease was the most frequent etiology, in particular, ACS in patients with good neurological outcome.

Another study demonstrated that unfavorable versus favorable neurological outcomes were associated with multiorgan failure syndrome on admission and during in-hospital stay, but independent predictors of unfavorable neurological outcome were MV on admission, high admission Simplified Acute Physiology Score (SAPS) II score, and neurological dysfunction on admission [24]. In our OHCA patients with ischemic brain injury, we observed significantly more likely the use of noradrenalin, dobutamine and MV, reflecting respiratory and cardiovascular failure in these patients. In addition, admission EF <35% was significantly associated with ischemic brain injury, reflecting acute systolic myocardial dysfunction in this subset of OHCA patients.

Involuntary muscular contractions/seizures were associated with ischemic brain injury as well. If present, EEG was recorded. In the case of epileptogenic EEG activity, antiepileptic drugs were added (levetiracetam IV, benzodiazepines IV, and barbiturates IV) until cessation to prevent secondary brain injury [10,11,13]. In addition, CT scans were performed to exclude or confirm morphological changes such as brain edema, bleedings, and localized or generalized brain hypoperfusion.

According to previous studies, good neurological outcome is more likely in early EEG reactivity, adequate EEG responses to auditory stimuli, and absence of abnormalities on CT/MR within 1 week of ROSC [10,11,13].

Several studies stressed the importance of underlying cardiac disease. In more than 50% of our OHCA patients, ACS was the underlying disease. ACS was more likely, though nonsignificantly, observed in patients with good neurological outcome in comparison to those with ischemic brain injury. However, good neurological outcome was more likely when PCI and dual antiplatelet therapy were used.

In addition, EF <35%, which was associated with ischemic brain injury, may have been the consequence of prior chronic cardiac disease and also acute systolic dysfunction or cardiogenic shock due to global myocardial hypoperfusion during OHCA, as well as of ACS and reperfusion injury in the post-resuscitation period.

Among the significant limitations of our study is its retrospective nature. However, the data originate from real life. We also observed that hypothermia of 32–34°C was not achieved in nearly 30% of OHCA patients – mainly due to early death, or early improvement of neurological status. However, therapeutic hypothermia was performed on the top of other recommended therapeutic strategies, aimed to optimize oxygenation, circulation, cardiac function, and early treatment of ACS. Our data confirmed the significant role of neurological outcome in the survival of OHCA patients.

## CONCLUSION

We conclude that early and optimal post-resuscitation care is important in improving the neurological outcome of OHCA patients and should include 24 hours of target temperature management – at least target temperatures <36°C. Our results did not confirm the significant role of hypothermia between 32°C and 34°C for the neurological outcomes, but did indicate the role of other therapeutic strategies such as the early treatment of ACS by PCI and dual antiplatelet therapy. We also confirm the role of older age, the cause of OHCA, cardiac dysfunction, and involuntary muscular contractions/seizures during in-hospital stay. Further studies are needed to elucidate the role of age in therapeutic hypothermia in OHCA patients.
